# Reversible, Electric-Field Induced Magneto-Ionic Control of Magnetism in Mesoporous Cobalt Ferrite Thin Films

**DOI:** 10.1038/s41598-019-46618-6

**Published:** 2019-07-25

**Authors:** Shauna Robbennolt, Enric Menéndez, Alberto Quintana, Andrés Gómez, Stéphane Auffret, Vincent Baltz, Eva Pellicer, Jordi Sort

**Affiliations:** 1 Departament de Física, Universitat Autònoma de Barcelona, E-08193 Cerdanyola del Vallès, Spain; 2 Institut de Ciència de Materials de Barcelona (ICMAB-CSIC), Campus UAB, E-08193 Cerdanyola del Vallès, Barcelona Spain; 3 SPINTEC, Univ. Grenoble Alpes/CNRS/INAC-CEA, F-38000 Grenoble, France; 4 Institució Catalana de Recerca i Estudis Avançats (ICREA), Pg. Lluís Companys 23, E-08010 Barcelona, Spain

**Keywords:** Chemical physics, Magnetic properties and materials, Magnetic properties and materials

## Abstract

The magnetic properties of mesoporous cobalt ferrite films can be largely tuned by the application of an electric field using a liquid dielectric electrolyte. By applying a negative voltage, the cobalt ferrite becomes reduced, leading to an increase in saturation magnetization of 15% (*M*_*S*_) and reduction in coercivity (*H*_*C*_) between 5–28%, depending on the voltage applied (−10 V to −50 V). These changes are mainly non-volatile so after removal of −10 V *M*_*S*_ remains 12% higher (and *H*_*C*_ 5% smaller) than the pristine sample. All changes can then be reversed with a positive voltage to recover the initial properties even after the application of −50 V. Similar studies were done on analogous films without induced porosity and the effects were much smaller, underscoring the importance of nanoporosity in our system. The different mechanisms possibly responsible for the observed effects are discussed and we conclude that our observations are compatible with voltage-driven oxygen migration (*i.e*., the magneto-ionic effect).

## Introduction

Magnetic materials overall are a very useful class of materials that have been used in many applications affecting our everyday lives. Over the past few decades, there has been a significant push towards miniaturization of devices which has been supported by the development of evermore precise techniques for controlling the micro- and nano-scale properties of materials. Traditionally in devices, magnetism has been controlled by the use of external magnetic fields created by passing current through a conducting wire. However, as these structures become smaller, resistive heating (or Joule heating) has made this method energetically too costly, leading to a significant research effort to control magnetic micro- and nano-structures directly with electric fields^1–5^.

A number of mechanisms for controlling magnetism using electric fields have been utilized. One approach has been to use multiferroic (MF) materials in which the magnetic moments are either directly coupled to electric dipoles (intrinsic multiferroics) or strain-coupled to piezoelectric materials in composite heterostructures^6–12^. There are very few intrinsic MF materials at room temperature, thus composite MF materials are more commonly used. However, these composites are currently limited by inefficient energy transfer between the electric and magnetic counterparts as well as mechanical failure over time due to the use of mechanical strain to couple the two components. Another approach has been to directly apply electric fields to metallic magnetic materials and take advantage of electrostatic surface charging and the concomitant induced changes in the 3*d* orbitals and the magnetic anisotropy energy. However, this approach is only effective for ultrathin films (<5 nm) because the electric field is screened at the very surface of the metal (within the Thomas-Fermi screening length)^1,4,5,13–18^. Alternatively, electric fields can be directly applied to magnetic oxide materials which can be electrically insulating, thereby avoiding electric field screening. In this case, five potential mechanisms through which the electric field can affect the magnetic properties can be mainly distinguished: MF coupling, electric charge accumulation and carrier density modulation, surface oxidation/reduction reactions, ion intercalation and other magneto-ionic effects.

MF coupling requires ferroelectric and magnetic components mutually coupled to each other, as described above. The system studied in this work does not contain a ferroelectric component, so MF coupling will not be further discussed here^6,7,19,20^. Charge accumulation, or the formation of the electric double layer (EDL), at the material surface can be seen in both solid-state and liquid electrolyte-gated configurations (particularly in oxide semiconductors). In solid-state systems, upon the application of a voltage, charges accumulate at the oxide-electrode interfaces which can locally change the orbital occupancies (modulating the carrier density in semiconductors) and consequently, the magnetic properties^1,14,21,22^. In liquid systems, an applied electric field induces the formation of an electric double layer in which ions in the liquid assemble at the material-liquid interface which can similarly affect the magnetic properties^18^. The drawback of charge accumulation is that it is only surface sensitive and is limited by the accessible surface area. Oxidation/reduction at the surface of the magnetic material caused by the applied electric field can also affect the magnetic properties. This redox process is known in the energy storage community as pseudocapacitance where there are charge transfer reactions at the interface between the electrolyte (generally liquid) and the active material. This requires transition metals that can switch between multiple, stable oxidation states. If the ions that are oxidized/reduced are magnetic, then the magnetic properties are affected by the change in oxidation state. For example, Fe^3+^ has 5 *µ*_*B*_, Fe^2+^ has 4 *µ*_*B*_, and Fe^0^ has only 2 *µ*_*B*_, so if everything else is kept constant, the sample magnetization will decrease as the Fe atoms become more reduced. It should be noted that surface oxidation/reduction is facilitated by having electrically conducting films, however, cobalt ferrite and other metal oxide films have been shown to exhibit pseudocapacitance as a result of either sufficiently thin films or percolation conductivity^23–26^.

Cation intercalation involves intercalating small ions, such as Li^+^ or Na^+^, into the active material as is done in energy storage systems like Lithium-Ion Batteries (LIBs). The magnetic material is placed in a solution containing the ion(s) to be intercalated and a voltage is applied which causes ion intercalation. For example, if a negative voltage is applied, Li^+^ ions are drawn to the active material and because they are sufficiently small, they are able to physically enter the crystal structure of the material either between layers if the material has a layered structure or by hoping between cation vacancies in structures such as spinel ferrites^27–30^. Ion intercalation causes an expansion of the crystal structure and can cause cation redistribution in the sample which can both affect the magnetic properties^23,25,26,31^. The drawbacks to this approach are that it is a relatively slow procedure and the insertion and removal of ions from the crystal structure can cause degradation and mechanical failure over time, often within a few cycles. Additional “magneto-ionic effects” can occur when the applied electric field causes ion migration (often O^2−^ migration) which in turn affects the magnetic properties^25,32^. Most work in the area of magneto-ionics has involved a metallic magnetic material which is oxidized with a positive electric field and then re-reduced using a negative electric field. This oxidation involves the migration of oxygen atoms from either an oxygen donor (*e.g*., HfO_2_ or GdO_2_), from a surface oxide passivation layer, or from a liquid electrolyte containing oxygen^32–40^. This approach can also be used to affect magnetism in metal oxides by causing migration of the oxygen that is already present, without the need for an external oxygen source. The use of a liquid electrolyte is typically the easiest method to apply voltage to these materials because solid oxygen donors like HfO_2_ involve a more complicated synthesis to add an additional solid layer. In addition, in solid state devices, it is difficult to avoid pinholes or defects which cause dielectric breakdown. However, liquid electrolytes do not suffer as much from dielectric breakdown which is why they are often used in proof-of-concept systems, like the one presented here. It is worth noting however that there is a growing interest in ionic gels which can be integrated into devices^41–43^.

In this work, we use a liquid electrolyte with a low concentration of ions to reversibly change the magnetism of cobalt ferrite (CFO) thin films through what appears to be a primarily magneto-ionic mechanism in which the oxygen ions (O^2−^) migrate in response to the applied electric field. Notably, the changes in magnetism of CFO observed here occur at room temperature without the need for additional heat. CFO is a widely studied material for a variety of applications due to its relatively high saturation magnetization (≈85 emu/g), chemical stability and ease of fabrication. It is an inverse spinel structure with the formula CoFe_2_O_4_. The oxygen atoms are arranged in a cubic close packed structure and, in theory, the Co^2+^ ions occupy octahedral (A) sites while the Fe^3+^ ions occupy the remaining octahedral (A) sites and the tetrahedral (B) sites. However, this is the theoretical cation distribution for an inverse spinel and often, in practice, the cation distribution can be less orderly due to defects (*e.g*., oxygen vacancies)^25,26,44–46^. Indeed, Chen *et al*. found that the cation distribution in oxygen-deficient non-porous CFO films, grown by pulse-laser deposition, can be locally modified by applying an electric field (and inducing cation migration) using a current atomic force microscope (C-AFM) tip. In this way, the authors were able to reversibly change the magnetic domain patterns at the nanoscale in the same region previously actuated using the C-AFM^47,48^.

Cobalt ferrite can be easily fabricated using sol-gel methods in which Co and Fe salts are dissolved in an oxygen-containing organic solvent where they form metal-oxide bonds. As more and more bonds develop over time, a network is developed, creating a gel structure. This gel can then be calcined to form cobalt ferrite powders or, alternatively, the solution before gelation can be deposited onto substrates to create thin films^30,49,50^. In this way, porous cobalt ferrite thin films can be synthesized by Evaporation Induced Self-Assembly (EISA)^50–52^. For example, Quickel *et al*. developed a procedure to use a block copolymer as a template to create porous cobalt ferrite^50^. Briefly, an amphiphilic diblock copolymer was added to the solution where it formed spherical micelles. The solution was then deposited onto a substrate by dip-coating and the micelles and metal oxide material self-assembled as the solvent was evaporated (EISA). The films were then heated to remove the polymer template and crystallize the sol-gel precursor into crystalline cobalt ferrite.

Here, we employ this same method to fabricate mesoporous thin films of CFO in order to investigate the magnetoelectric behavior of the resulting material as a whole (not locally as in ref.^48^). We apply the electric field using a liquid electrolyte in order to fully access all of the open porosity of the film. The electrolyte (Na^+^, OH^−^) concentration was kept low (5 ppm) in order to minimize ion intercalation into the cobalt ferrite. This low electrolyte concentration also allows us to apply higher voltages without detrimental solvent degradation as is often seen in battery systems, which typically use electrolyte concentrations between 1 M–0.1 M. By using an electrolyte concentration that is over 10,000 times smaller, we significantly limit the cell current which in turn should limit the solvent degradation rate. In this case, much of the propylene carbonate solution can be seen as acting more as a dielectric material than a current conductor. We find large changes in the saturation magnetization (*M*_*S*_) and coercivity (*H*_*C*_) of the nanoporous films with applied negative voltage (measured using a macroscopic magnetometry technique) that are completely reversible with the application of a positive voltage. We find through X-Ray Photoelectron Spectroscopy (XPS) and X-Ray Diffraction (XRD) studies that applying a negative electric field increases the amount of zero-valent or metallic Co^0^ and Fe^0^ and the average lattice parameter of the cobalt ferrite decreases. This suggests that the negative electric field is causing oxygen ion migration to the surface of the material which is responsible for the increased *M*_*S*_ and decreased *H*_*C*_ that is observed. When a positive electric field is then applied, these changes are reversed and the amount of zero-valent Co^0^ and Fe^0^ decreases while the lattice parameter increases. This suggests that the oxygen ions migrate back from the surface to the material interior which is again consistent with the observed changes in the magnetic properties. Interestingly, the same investigations were done on thin films made without the polymer template (*i.e*., more dense films) and we found that the magnetic properties did change, but to a much lower degree. Furthermore, we found that the electronic resistivity of the films decreased by almost two orders of magnitude after the application of −10 V and then increased again after the application of +10 V. This work constitutes a clear step further towards effective magneto-ionic control of the magnetic properties of oxide thin films, previously accomplished only in some other complex oxides (*i.e*., perovskites), at temperatures below room temperature^53–56^.

## Materials and Methods

### Materials

Cobalt(II) nitrate hexahydrate (99.99%), iron(III) nitrate nonahydrate (99.999%) and 2-methoxyethanol (99.8%) were purchased from Sigma-Aldrich. Poly(ethylene oxide)-b-Poly(butylene) with Mn: PEO(4800)-PB(5800), was obtained from Polymer Source. All chemicals were used without further purification.

### Preparation of the Sol-Gel solutions

The initial sol-gel solution was prepared following a previously published recipe^50^. Co(NO_3_)_2_·6H_2_O (220 g) and Fe(NO_3_)_3_·9H_2_O (620 mg) were dissolved in 2 mL of 2-methoxyethanol and 2 mL of ethanol. This solution was mixed using magnetic stirring at room temperature overnight and was never found to be cloudy at this point. Simultaneously, 10 mg of PEO-PB was dissolved in 1 mL of ethanol and allowed to stir overnight. The next day half of the solution containing the dissolved metal salt was added to the dissolved polymer and then 1 mL of ethanol was added to the remaining half so that each solution was a total volume of 3 mL. The solution without the polymer was used to make the non-templated films while the one with polymer was used to make the porous films.

### Thin film deposition

The substrates were prepared by sputtering 70 nm of Pt onto wafers of Si (100). The wafers were then cut into 3 cm × 1 cm pieces for the dip-coating process. The back of the substrate was taped to avoid the deposition of CFO on the back of the chips. The solution was deposited onto the chips by dip-coating with a withdrawal rate of 300 mm/min. The films were immediately transferred to a hot-plate at 80 °C for 10 min to aid the evaporation of the solvent and avoid the absorption of too much water from the air. The films were heated in air to 600 °C at a rate of 3 °C/min and held at 600 °C for 3 h before being allowed to cool to room temperature. The dense and porous films were prepared and calcined together in order to offer the best comparison between the films. Both sets of films were 90–95 nm thick.

### Magnetic characterization

**Figure 1:**
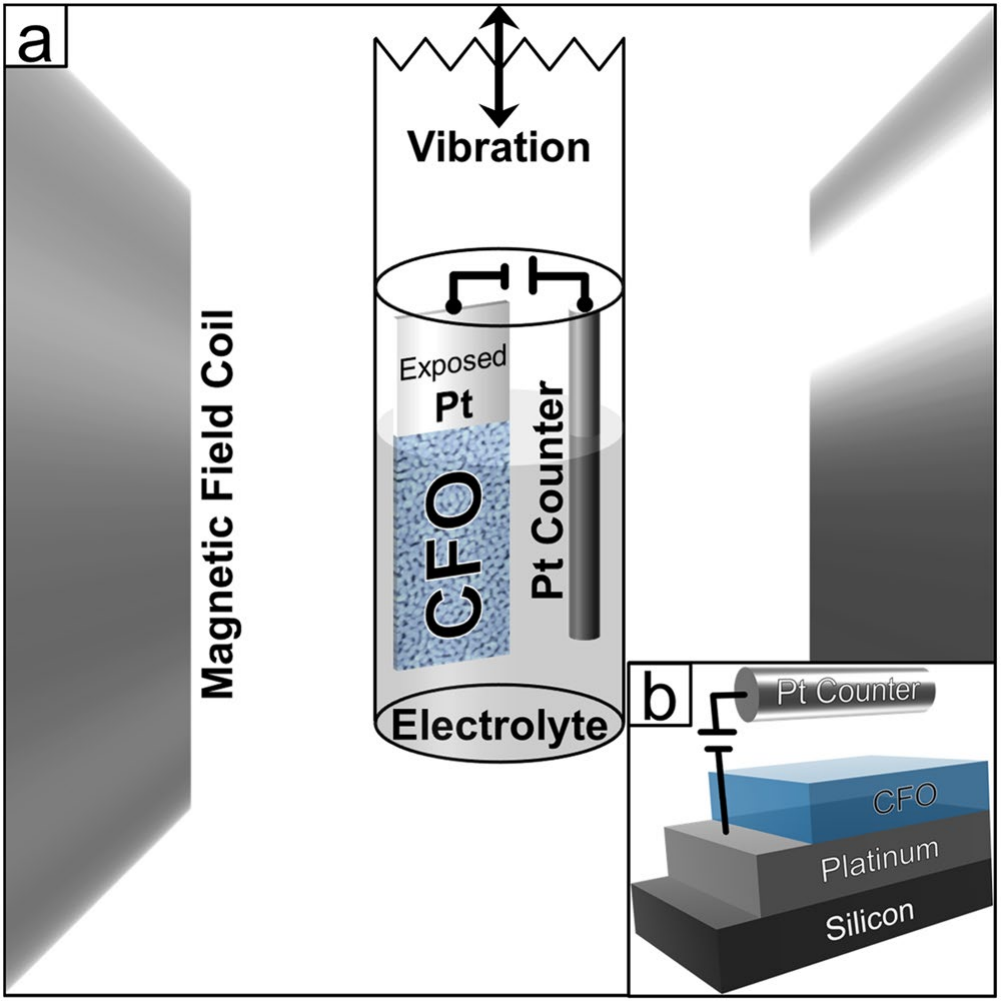
Schematic of the cell built for applying an electric field *in situ* while measuring the magnetic properties in a Vibrating Sample Magnetometer. The electrochemical cell contained the CFO sample as the working electrode and a Pt wire counter electrode. The working electrode contact was made to the Pt underneath the CFO sample. The electrolyte was propylene carbonate with a small amount of dissolved NaOH. The whole cell was attached to a VSM holder and vibrated vertically between the magnetic field coils. The inset shows the layers of the sample from a side view with the Pt counter electrode.

The magnetic properties were measured using a MicroSense (LOT-QuantumDesign) Vibrating Sample Magnetometer (VSM). The voltage was applied using an Agilent B2902A power supply. Figure 1 shows a schematic of the electrochemical cell that was built to be used *in situ* during VSM measurements. Since the films were prepared by dip-coating, there was a small area where the substrate was held that was not coated by the cobalt ferrite and therefore remained exposed platinum. This area was used to make the electric connection to the sample, *i.e*., by indium welding a Cu wire to the exposed Pt. A Pt wire was used as the counter electrode which was then held at a constant distance from the sample using a hot-melt polymer adhesive. The sample was then placed in a small Eppendorf tube and filled with propylene carbonate treated with metallic sodium to remove any traces of water which left a small amount of Na^+^ and OH^−^ ions (≈5 ppm Na^+^ as determined by Inductively Coupled Plasma (ICP) Spectroscopy). All magnetic measurements were taken in an in-plane configuration at room temperature.

### Structural, elemental and electrical characterization

Field Emission Scanning Electron Microscope (FE-SEM, Zeiss Merlin) with Energy Dispersive X-ray Spectroscopy (EDX) capabilities was used to characterize the microstructure of the films and the elemental composition. Grazing Incidence X-Ray Diffraction (GIXRD) measurements (2θ scan) were performed in a Materials Research Diffractometer (MRD) from Malvern PANalytical company. This diffractometer has a horizontal Ω-2θ goniometer (320 mm radius) in a four-circle geometry and it works with a ceramic X-ray tube with Cu K_α_ anode (λ = 1.540598 Å). The detector used is a Pixcel which is a fast X-ray detector based on Medipix2 technology. The crystallographic structural parameters, such as lattice parameter or crystallite size, have been evaluated by fitting the GIXRD patterns in the 32–40° 2θ range using the Materials Analysis Using Diffraction (MAUD) Rietveld refinement program^57–59^. XPS analyses were performed using a PHI 5500 Multitechnique System (Physical Electronics) with a monochromatic Al K_α_ source under ultra-high vacuum. All XPS scans were carried out on the film surface. The spectra were calibrated using the carbon peak that arises from “adventitious” carbon on the film after exposure to air. The GIXRD patterns and XPS spectra were performed *ex situ*. In these cases, the samples were put into an electrochemical cell identical to that described above for the magnetic characterization for the application of voltage and then the samples were removed, and the measurements were performed. For GIXRD measurements the samples were subjected to the indicated voltage for 1 h before the measurement and for the XPS measurements, the samples were soaked for the indicated time at the indicated voltages. Conductive Atomic Force Microscopy was used to investigate the electrical properties. These measurements were performed using a Keysight AFM 5500 LS using an external current-to-voltage amplifier module “Resiscope”. To acquire the data, a full metallic tip with part number “RMN-25PtIt300b” from Rocky Mountain Nanotechnology was used, while a constant bias of 9 V is applied to the sample. The images were performed at a constant speed of 21.7 μm/s while a force of ≈2 μN was used for all the scans. The spectroscopy IV curves were performed with a slew rate of 4 V/s. All the experiments were performed under low humidity, less than 8% to avoid possible electrochemical artifacts.

## Results and Discussion

**Figure 2:**
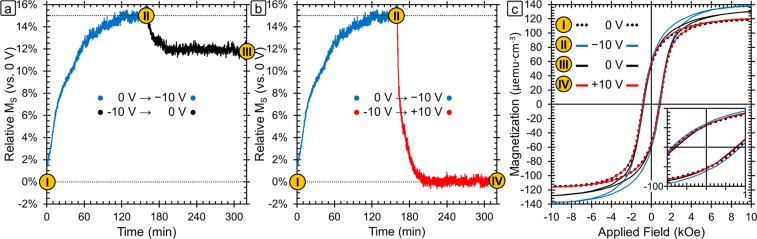
(**a**) Relative change in saturation magnetization (*M*_*S*_) vs. the initial sample magnetization as a function of time and applied voltage. Point ***I*** is the initial sample as-synthesized. The blue line shows the evolution of the magnetization during the application of −10 V for 160 mins and point ***II*** is the maximum magnetization reached. After the application of −10 V, the voltage was turned to 0 V (black line) in one case and +10 V (red line) in another. Points ***III*** and ***IV*** are the sample state after the sample relaxed at 0 V and recovered under +10 V respectively. (**b**) Room-temperature magnetic hysteresis loops corresponding to points ***I***–***IV***.

Figure 2a,b shows the relative change in saturation magnetization over time as a function of voltage. The *M*_*S*_ of the sample in the initial state (0 V) is used as the baseline (0%) and the percent change is plotted relative to that value. For these measurements, the field was fixed at 15 kOe which is much higher than the field required to saturate the sample. Hysteresis loops at various points of interest are presented in Fig. 2c and those points are marked in Fig. 2a,b. The hysteresis loop of the initial state (0 V) is in black dotted lines and corresponds to point **I**. The first voltage applied was −10 V, as shown in blue, and the sample magnetization is found to increase until it reaches a saturation point after 120 min at 15% higher than the initial state. The timescale of the observed changes is too long to be adequately explained by charge accumulation alone since charge accumulation happens typically in seconds. Ion intercalation has been shown by Dubraja *et al*. to increase *M*_*S*_ in cobalt and nickel ferrites^30^. In that work the authors explain that the intercalated Li^+^ ions reduce the Fe^3+^ ions in tetrahedral sites to Fe^2+^ which leads to the increase in the sample magnetization. This is because magnetism in spinel ferrites is based on exchange interactions between ions in the octahedral and tetrahedral cation sites. The net magnetic moment is determined by the excess of unpaired electrons in the octahedral sites, so increasing unpaired electrons in octahedral sites or decreasing them in tetrahedral sites causes increased ferrimagnetism^24,30,57^. However, typically the change in magnetization observed due to ion intercalation is lower (<5%) than has been observed here (15%). The oxidation/reduction and magneto-ionic effect mechanism can also cause similar changes resulting in an increase in *M*_*S*_. In particular, the latter mechanisms can cause metallic counterparts (like clusters) to form during reduction which could also lead to an increase in *M*_*S*_ and decrease in *H*_*C*_. Next, +10 V was applied to the sample (red line) and the magnetization is found to return completely to the initial value after ≈40 min (Fig. 2b). Even after longer time periods at +10 V, the magnetization does not go below the initial value. Furthermore, the hysteresis loop at point **IV** (red) shows that the *H*_*C*_ returns to the initial state as well.

In order to explore the effect of sample history, we ran the same experiment on a pristine sample but applied the positive voltage first and found that there was no change upon the application of +10 V. This asymmetric response with respect to the voltage polarity is consistent with ion intercalation since, due to their smaller size, the Na^+^ ions are more likely to intercalate than the OH^−^ ions and Na^+^ intercalation happens at negative voltages. It is possible that oxidation/reduction reactions play a role and that the Co and Fe atoms start off at the highest stable oxidation state so that the application of a positive voltage would cause no further changes. However, the XPS results discussed below suggest that this is not the case. Finally, our results are consistent with the magneto-ionic effect which would involve oxygen ions to migrate into the sample under positive electric fields. In this case, there is very little oxygen in the electrolyte, so there would be no oxygen available to migrate into the sample until after a negative voltage has already been already applied. This would again account for the voltage-asymmetry in the obtained magnetoelectric results.

The hysteresis loops taken at points **II** and **IV** were recorded while −10 V and +10 V were applied respectively. In addition, we explored the stability of the state with the highest *M*_*S*_ achieved after the application of −10 V. For this purpose, we applied −10 V in the same way as before to reach point **II** and then set the voltage back to 0 V and measured the magnetization over time again (point **III**, black) (Fig. 2a). The magnetization decreased and leveled off at a 12% higher value than in the as-prepared state (−3% decrease with respect to −10 V). It should be noted that from point **III** the application of a positive voltage returns the magnetization to point **IV**, meaning that both cycles (−10 V, +10 V, 0 V) and (−10 V, 0 V, +10 V, 0 V) result in the same end point. This behavior is favorable for many applications since it means it is not necessary to be continuously applying the negative voltage to maintain a stable state with increased magnetization (*i.e*., non-volatile behavior). Moreover, the change in magnetism is entirely reversible with the application of a positive voltage. From a mechanistic standpoint, we would expect that “pure” charge accumulation effects would be completely reversible, hence indicating that other mechanisms are needed to fully explain the data. The other three mechanisms (ion intercalation, surface oxidation/reduction reactions, and magneto-ionic effects) could indeed result in a largely irreversible behavior in the absence of an applied positive electric field, as observed experimentally.

**Figure 3:**
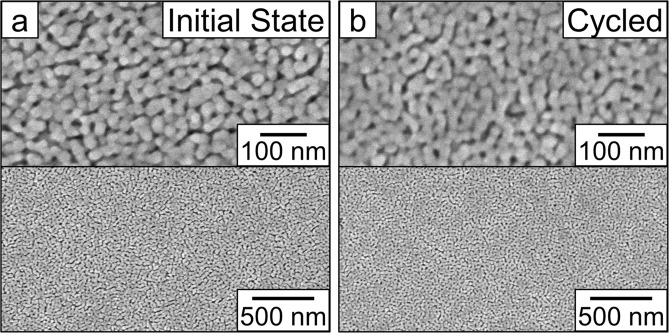
Top-view scanning electron microscopy (SEM) images of the porous cobalt ferrite film (**a**) as prepared in the initial state (left) and (**b**) after one cycle of 160 min at −10 V followed by 160 min at +10 V (right). The top images are at a higher magnification to show the pore structure and the bottom images are the same film more zoomed out to demonstrate the homogeneity.

Figure 3 shows top-view SEM images of the film in the initial state (0 V, point **I**) and after one voltage cycle which is the application of −10 V for 160 min followed by the application of +10 V for 160 min (point **IV**). In both cases, the SEM images were taken at remanence with no applied electric field during the measurement. The images were acquired both at a high magnification (top), to show the porous structure, and at a lower magnification (middle), to show the homogeneity of the films over a larger area. The images show a porous structure with ligaments around 20 nm in diameter. The observed morphology is worm-like and similar to that found in the initial publication of this synthesis by Quickel *et al*. as well as by others who have used the same procedure^30,50,60,61^. Importantly, the film morphology is found to be maintained after the application of a negative and positive voltage. The image of the cycled film was a bit more difficult to resolve, likely due to a layer of organic solvent remaining after the electrochemical treatment which was difficult to completely remove.

**Figure 4:**
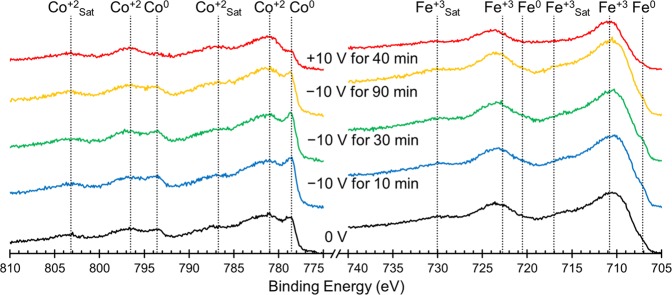
Elemental XPS spectra for Co (left) and Fe (right) ordered chronologically from bottom to top starting with the initial state at 0 V (black lines) followed by spectra taken after the application of ‒10 V for 10 min (blue lines), 30 min (green lines) and 90 min (yellow lines) and finally after the application of +10 V for 40 min (red lines). The peaks are assigned based on previously published peak assignments found in the NIST XPS database.

To better understand the underlying mechanism that causes the observed change in magnetism, we probed the oxidation states of the Co and Fe surface atoms using XPS and the resulting spectra are presented in Fig. 4. The sample was cycled in the same way as above, starting with an initial measurement at 0 V (black lines, bottom) and then spectra were taken after the application of −10 V for 10, 30 and 90 min (blue, green, yellow lines, respectively) and finally after the application of +10 V for 40 min (red lines). Table 1 shows the percentage of each element that is present in the metallic state as calculated using peak areas. In the initial state (0 V, black lines) the Co is found to be in a mixture of Co^2+^ and Co^0^ (7.8%) while the Fe is found to be mostly oxidized in the form of Fe^3+^ with only 0.6% of Fe being Fe^0^. In theory, cobalt ferrite should not have any zero-valent (metallic) Co or Fe, however experimentally, spinel ferrites are prone to oxygen deficiencies which lead to reduced metallic species^24,60^. After 10 min at −10 V, the amount of metallic Co increases to 9.8%, tending to level off at this value. These values of Co^0^ percentages for different times at −10 V are all within error of each other which suggests that the Co on the top of the film is reduced almost immediately after the application of the negative voltage. The Fe on the other hand increases only slightly after 10 min at −10 V, from 0.6% to 0.8% metallic, but increases further to 1.5% and 1.3% after 30 and 90 min respectively. This suggests that the Fe on the top of the film takes longer to become maximally reduced. This is likely because Fe^3+^ undergoes a two-step reduction in which Fe^3+^ reduces first to Fe^2+^ which can then be reduced to Fe^0^, as opposed to the reduction of Co which proceeds directly from Co^2+^ to Co^0^.

**Table 1:** The percentage of each element that is found to be metallic (oxidation state of 0).

State	Co^0^	Fe^0^
−00 V for 00 min	7.8%	0.6%
−10 V for 10 min	9.8%	0.8%
−10 V for 30 min	9.9%	1.5%
−10 V for 90 min	9.6%	1.3%
+10 V for 40 min	2.2%	0.2%

For Co, these values were determined by calculating the area under the Co^0^ peak at 778.6 eV and the Co^2+^ peak at 781.0 eV and calculating what percentage of the total area was Co^0^. For Fe, these values were determined by calculating the area under the Fe^0^ peak at 707.1 eV and the Fe^3+^ peak at 710.8 eV and calculating what percentage of the total area was Fe^0^.

The film is found to be in the most oxidized state after the application of +10 V for 40 min, after which only 2.2% of Co and 0.2% of Fe on the top of the film remain metallic. There are two potential causes for the films having less zero-valent Co and Fe after a voltage cycle than in the initial XPS spectrum. One is that since the sample is immersed in a propylene carbonate solution during the application of the electric field, it might be possible that a layer of solvent remains on the film after the cycling which can change the XPS results somewhat. The second potential cause is that when electric fields are applied to the film, the changes in the film occur first at the top of the film and then move from the surface deeper into the film. If that is the case, then the initial XPS spectra was likely representative of the entire film while after a voltage is applied, the top of the film is likely different from the bottom of the film and is less representative of the entire film. This would explain why after −10 V is applied, there is no statistically relevant change in the amount of zero-valent metal ions over time. XPS is surface-sensitive and so if the top of the film is reduced first, the XPS would show that change and then level off. In contrast, VSM is sensitive to the entire sample; hence, so as the reduction process penetrates further into the sample, changes in magnetization over time are observable. Similarly, after the application of a positive voltage, the top of the film would oxidize first leading to a lower zero-valent ion content observed in XPS after the positive electric field was applied than was observed initially.

These results are consistent with the three aforementioned possible mechanisms: ion intercalation, oxidation/reduction and the magneto-ionic effect. Since the XPS study done here was done *ex situ* and the electric double layer is only present during the application of the electric field, we cannot say anything about potential charge accumulation from these results, although the dynamics of the system (Fig. 2) seems to rule out this mechanism. While a very low amount of Na^+^ and OH^−^ ions are present in the solution (5 ppm, as determined by ICP analysis), Na^+^ intercalation during negative voltage application and then deintercalation upon positive voltage application is consistent with these results. Na^+^ intercalation could cause reduction of Co and Fe within the sample as is seen when −10 V is applied and then upon the application of +10 V, Na^+^ deintercalation could cause reoxidation, as is seen in the XPS. It is also possible that the reduction and then oxidation of the Co and Fe is unaccompanied by ion movement and is caused by pseudocapacitance, which has been previously observed in cobalt ferrite (although typically on the millisecond timescale)^29^. Finally, the initial presence of Co^0^ and Fe^0^ is attributed to oxygen vacancies and the increase of these zero-valent species could result from further O^2−^ migration, consistent with the magneto-ionic effect^24^.

**Figure 5:**
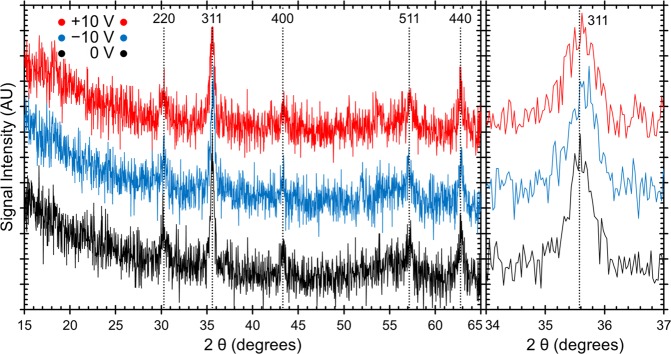
Grazing Incidence X-Ray Diffraction patterns for the sample at 0 V (black, point **I**), −10 V (blue, point **II**), and +10 V (red, point **IV**). The largest peak (311) is shown zoomed in on the right side in order to better show the slight observed shift.

In order to narrow down the possible mechanisms at work here, the crystal structure was probed by Grazing Incidence X-Ray Diffraction (GIXRD). Figure 5 shows the GIXRD patterns for the film in the initial state (black), after the application of −10 V (blue) and then after the application of +10 V (red). Only spinel cobalt ferrite peaks are observed with no other phases detected. The right side of the figure is zoomed into the largest peak (311). There are slight peak shifts in all of the peak positions, and this shift is most clear in the (311) peak since it is the one with the highest intensity. From the shift in the peak position, we can then calculate the lattice parameter (a) using Rietveld analysis^57–59^. We find a to be 8.367 ± 0.002 Å at 0 V; after −10 V it decreases to 8.357 ± 0.002 Å and then after +10 V it increases back to 8.369 ± 0.002 Å. If the primary mechanism were Na^+^ intercalation, we would expect the crystal lattice to either expand or stay the same with a negative electric field since the structure would have to accommodate the new Na^+^ ions. Similarly, if the mechanism was purely oxidation/reduction, we would expect no change in lattice parameter since no ions are physically migrating and since oxidation/reduction generally happens only at the surface whereas XRD probes the entire material. The decrease in lattice parameter is however consistent with O^2−^ migration from the bulk of the material to the surface^62^ or to the electrolyte^63^. It has been shown that oxygen ions can be dissolved into liquid electrolyte solutions in the form of Na_2_O or dissolved O_2_^63^. However, if the migratory oxygen ions were dissolved into the solution, over time they would diffuse and when a positive electric field was applied, diffusion kinetics dictates that not all of the diffused oxygen would be reincorporated into the cobalt ferrite. However, from the magnetization data, we see that the process is not only entirely reversible, but that the recovery of the initial state happens *faster* (40 min) than the initial change (120 min) as shown in Fig. 2a. From this, it seems that it is more likely that the migratory O^2−^ ions are accumulating at the material surface or at grain boundaries when a negative electric field is applied and then returning to the bulk of the material upon the application of a positive voltage. From the same GIXRD data, we find that the average crystallite size is 30 ± 2 nm at 0 V, decreases to 25 ± 3 nm after the application of −10 V and then further decreases to 21 ± 5 nm after the application of +10 V (this is already obvious from the peak broadening in the inset of Fig. 5). The initial decrease in crystallite size is consistent with the magneto-ionic mechanism since oxygen removal should shrink the size of the domain that can coherently diffract, which is what is determined here. Theoretically, we would expect that after applying a positive voltage, the crystallite size would increase back to the initial size. However, experimentally, it is likely that the oxygen migration back into the bulk of the material either crystallizes differently than the existing domain and therefore would not diffract coherently or the oxygen migration creates defects or amorphous regions which limit the calculated domain size as well.

**Figure 6:**
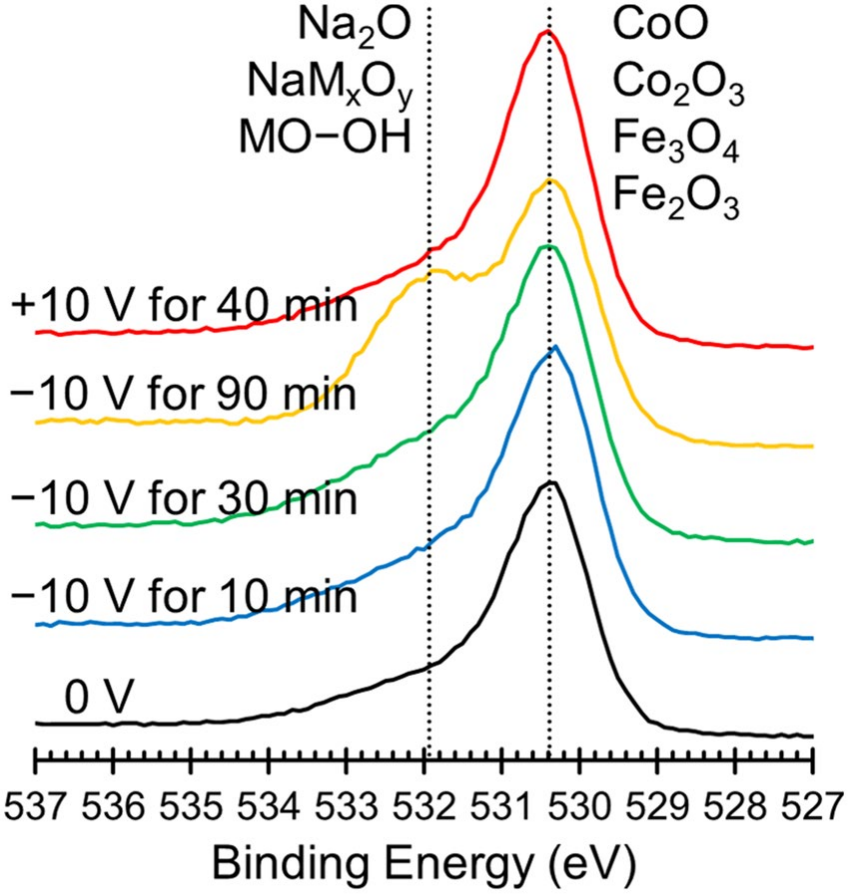
Elemental XPS spectra for O ordered chronologically from bottom to top starting with the initial state at 0 V (black lines) followed by spectra taken after the application of −10 V for 10 min (blue lines), 30 min (green lines) and 90 min (yellow lines) and finally after the application of +10 V for 40 min (red lines). The peaks are assigned based on previously published peak assignments found in the NIST XPS database.

Figure 6 shows the oxygen elemental XPS spectra that was collected at the same time as the XPS spectra in Fig. 4. The primary oxygen peak corresponds to metal oxides, which is what we would expect from this sample. At a higher binding energy, a peak corresponding to either Na_2_O, NaM_*x*_O_*y*_, or metal hydroxides, (here M denotes either Co or Fe) or metal hydroxides grows in during the application of −10 V and is most pronounced after 90 min at −10 V (yellow line). This peak then disappears after the application of +10 V for 40 min (red line). This data suggests that O^2−^ ions do indeed migrate in response to an electric field. Unfortunately, all of these species are too close in binding energies to differentiate, so we cannot say with certainty if the oxygen is stored on the surface as hydroxides or in solution as Na_2_O. Due to the complete reversibility of the system, we suspect that most of the oxygen in stored as surface hydroxides although it is likely that some dissolves into the electrolyte to form Na_2_O.

**Figure 7:**
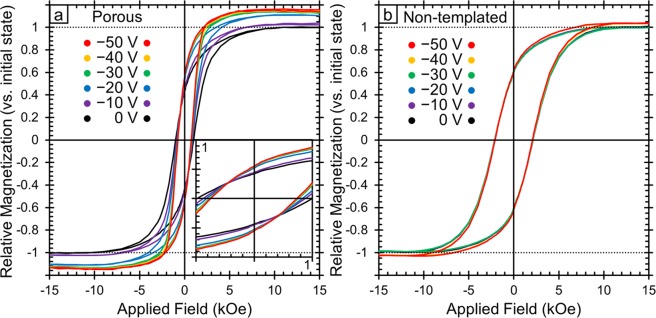
Room temperature magnetic hysteresis loops for both the porous (**a**) and non-templated (**b**) films as a function of applied voltage. In this case, the voltage was applied for 20 min and then the hysteresis loop was measured for 20 min leading to a total time of 40 min between each loop. To compare between the samples, the saturation magnetization (M_S_) was normalized such that the initial state (0 V) was set to 1. The inset in panel **a** shows the region near zero applied field in order to better observe the changes in coercivity.

The role of porosity in contributing to the large changes in magnetic properties that we observe was explored by preparing a set of films in the same way (and at the same time) but without the addition of the block copolymer templating agent that was used to create mesopores. Figure 7a shows the *M*-*H* loops of the porous films as a function of applied voltage and Fig. 7b shows the same data for the non-templated films. Here, the voltage was applied to the sample and left for 20 min before the measurement began. The voltage was maintained during the measurement which took 20 min leading to a total of 40 min between the measurement of each loop. In order to better compare between the two films, the sample magnetization was normalized in each case by setting the *M*_*S*_ at 0 V to be 1. The sample magnetization of the non-templated film was found to increase as progressively more negative voltages were applied, but not to the same extent as the porous film. The maximum change in *M*_*S*_ for the non-templated film is found after −50 V to be 2% higher than the initial value as opposed to the 15% increase observed in the porous film. (It is worth noting that in the porous film, a 15% increase in *M*_*S*_ was also shown with the application of −10 V, but only after >150 min whereas here, 15% increase is seen after only 40 min at −50 V). Furthermore, the *H*_*C*_ of the non-templated film is found to change only by 4%, even after the application of −50 V, whereas the porous film shows a coercivity decrease of 28% at −50 V. This demonstrates the importance of the mesoporosity in the templated sample to affecting magnetism with an applied electric field. The mesoporosity promotes larger magneto-ionic effects possibly due to two main reasons: (i) it enhances the overall surface area of material in contact with the electrolyte, (ii) it allows for larger electric fields since the distance between the Pt seed layer (which is electrically charged during magnetoelectric actuation) and the electric double layer that forms around CFO is significantly smaller in the nanoporous films (since the electrolyte penetrates towards the interior of the pores and the ligament size is around 20 nm) than for the dense CFO film (which is approximately 90 nm thick).

**Figure 8:**
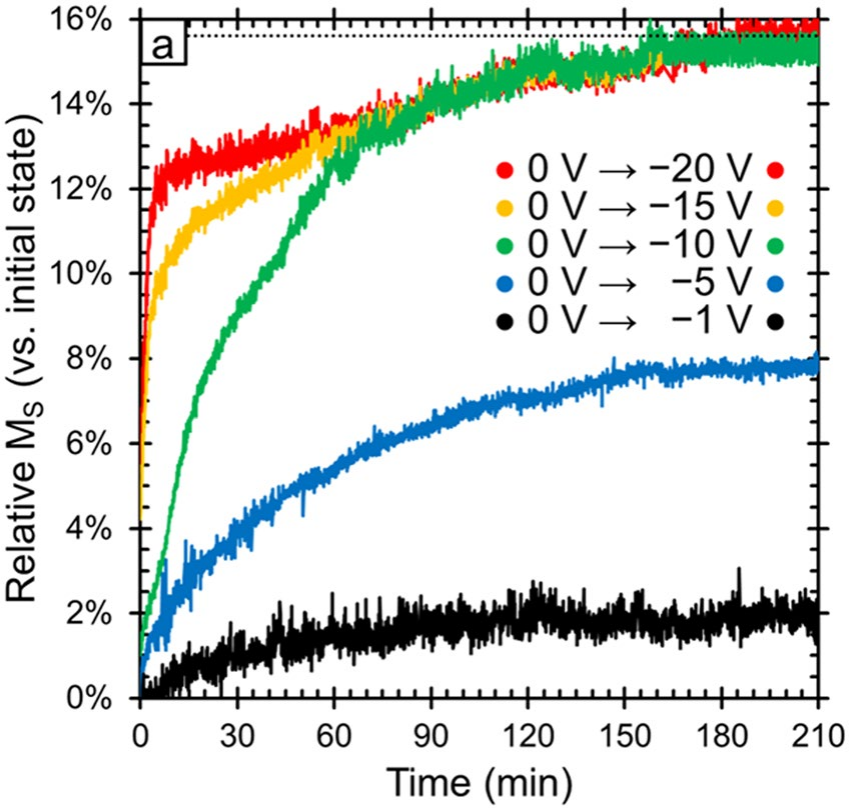
Relative change in saturation magnetization (M_S_) vs. the initial sample magnetization as a function of time and applied voltage. In each case, +10 V was applied for the same amount of time (210 min) in order to reverse the changes and in all cases, the sample magnetization returned to the initial state (these curves are omitted for the sake of clarity).

Figure 8 shows the evolution of the sample magnetization in a manner analogous to Fig. 8a, but in this case showing the effect of different negative voltages. We find that higher negative voltages lead to much faster kinetics. For example, when applying −20 V, the sample magnetization increased by 12% in within the first 5 min whereas that level of increase was only achieved after 1 h at −10 V. However, given enough time, the sample magnetization reaches a maximum after a 15% increase and then levels off for applied voltages of −10, −15 and −20 V. This shows that applying stronger negative voltages causes the reduction process to happen faster, but the material reaches a natural limit above which no further changes to *M*_*S*_ can be induced. (It is worth noting that the sample magnetization reaches the same maximum when larger voltages, up to −50 V are applied as well.) Smaller negative voltages showed much slower changes in *M*_*S*_, as is expected. These findings are consistent with the ion intercalation and magneto-ionic effect mechanisms but are not well explained by charge accumulation. First, the time scales involved here are fairly long (minutes-hours) which is more on par with ion migration (either Na^+^ or O^2−^) whereas effects from charge accumulation should be much faster. This is well documented in the energy storage field where capacitors (which use charge accumulation) are known to be much faster at charging/discharging than Li^+^ or Na^+^ ion batteries, which are notoriously slow. Furthermore, we find that smaller voltages (*e.g*., −1 or −5 V) plateau at lower sample magnetizations suggesting that between −1 and −10 V, the steady-state or long-term magnetization can be tuned by changing the magnitude of the voltage applied. This behavior on its own can be interesting for applications where having multiple accessible levels is beneficial such as neural-network-inspired computing. This also suggests that the mechanism is not simply oxidation/reduction because those reactions take place at discrete potential windows dictated by the reduction potentials of the elements involved with some flexibility due to kinetic limitations (electronic conductivity of the electrons or the need for overpotentials to overcome activation energies). This means that oxidation/reduction reactions would have a critical voltage below which they would not happen, but above that voltage, the processes would occur and given sufficient time, reach the same ending point. In this case, stronger applied electric fields would only affect the rate at which the processes occur, but would not result in the lower finals magnetizations found in the cases of −1 V and −5 V. That is not to say that charge accumulation or oxidation/reduction does not contribute, but from the kinetics data in Fig. 8 it is clear that these mechanisms are insufficient on their own to explain our results.

**Figure 9:**
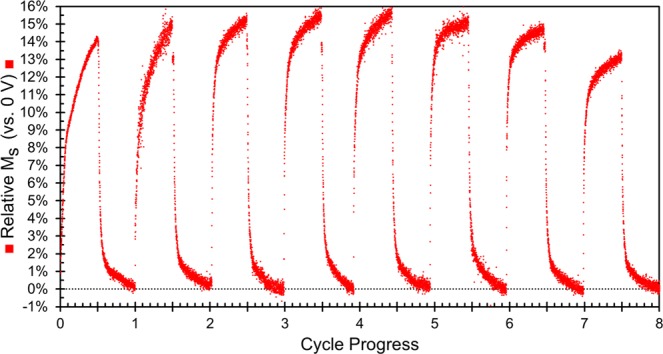
Change in sample magnetization (vs. the initial state) measured at 15 kOe. Each cycle took a total of 180 min: 90 min at −10 V followed by 90 min at +10 V. The first 8 cycles are shown because one of the electrodes disconnected during the 9^th^ cycle.

The data discussed thus far has focused on how the initial sample changes during the first cycle of −10 V followed by +10 V. In order to better understand the reversibility of the observed changes, we measured the magnetization while cycling a sample for 90 min at −10 V then 90 min at +10 V. Figure 9 shows the relative change in magnetization vs. the initial state, at a magnetic field of 15 kOe. During the first cycle, the sample magnetization reaches a maximum at ≈14% higher than the initial state during the application of −10 V and then returns to the initial value after the application of +10 V which is in good agreement with Fig. 1. Over the first 5 cycles, the maximum sample magnetization reached continues to increase and reaches a maximum of a 15% increase vs. the original value. This is also consistent with Fig. 8 where the *M*_*S*_ levels off after reaching a 15% increase. After the fifth cycle, the maximum magnetization reached begins to decrease and by the 8^th^ cycle, the maximum magnetization reached is below that of cycle 1, but still larger than 13%. This is again consistent with ion intercalation and magneto-ionic effects. This type of behavior has been previously reported for cobalt ferrite subjected to Li^+^ intercalation where the electrochemical capacity and sample magnetization were both observed to fade as the material was cycled^30^. However, in that work, much higher concentrations of Li^+^ were used to ensure that ion intercalation was the primary mechanism which led to the fading within 3 cycles (likely due to material or electrolyte degradation). Here we used a much lower ion concentration which should be “gentler” and is likely the reason we are able to cycle the material 8 times and still maintain a 13% increase in *M*_*S*_.

From this set of data, it seems that the primary mechanism through which the applied electric field tunes the magnetic properties is the magneto-ionic effect with a potential contribution from ion intercalation and, to a lesser extent, “pure” charge accumulation and oxidation/reduction. Charge accumulation effects are difficult to assess in this study because both the XPS and GIXRD studies had to be performed *ex situ* due to the nature of the experimental setups. However, the fact that changes in the oxidation states and lattice parameters were observed by these techniques, respectively, suggests that at least one other mechanism is involved. This is further supported by the data in Fig. 2a where we can see that when the negative electric field is removed and set to 0 V, the process is mostly irreversible whereas charge accumulation effects are expected to be entirely reversible. Finally, the time scale of the change in magnetism shown in Figs 2a and 8 is too slow and the magnitude of the change is too large for charge accumulation alone.

Pure oxidation/reduction reactions are also potentially present in our system, especially since pseudocapacitance has been seen before in cobalt ferrite^29^. This mechanism is consistent with the XPS data (Fig. 4) but, like charge accumulation, oxidation/reduction processes are too fast to explain the slow kinetics observed in our system. Furthermore, oxidation/reduction without any type of ion migration does not explain the change in *d*-spacing with applied electric field observed in GIXRD (Fig. 5). Ion intercalation, on the other hand, is a slower process and is more in line with the kinetics results. It can also explain the observed changes in the oxidation states of Co and Fe seen in the XPS spectra and the decrease in maximum *M*_*S*_ found in the cycling data in Fig. 9. However, like charge accumulation and oxidation/reduction, ion intercalation cannot explain the observed change in *d*-spacing.

The magneto-ionic effect in which O^2−^ ions migrate in response to the applied electric field is the only mechanism that is consistent with all of the results presented here, including the GIXRD. In this material, the oxygen atoms are the largest in terms of volume and oxygen migration away from the bulk of the material would lead to a decrease in the lattice parameter (*d*-spacing). Previous reports have shown that oxygen can aggregate at grain boundaries or at the material surface or be dissolved into the electrolyte^39,63^. In any of these cases, the oxygen that migrated would not likely take a crystalline form that can be detected by XRD which is the reason that no other species are observed in the GIXRD patterns.

**Figure 10:**
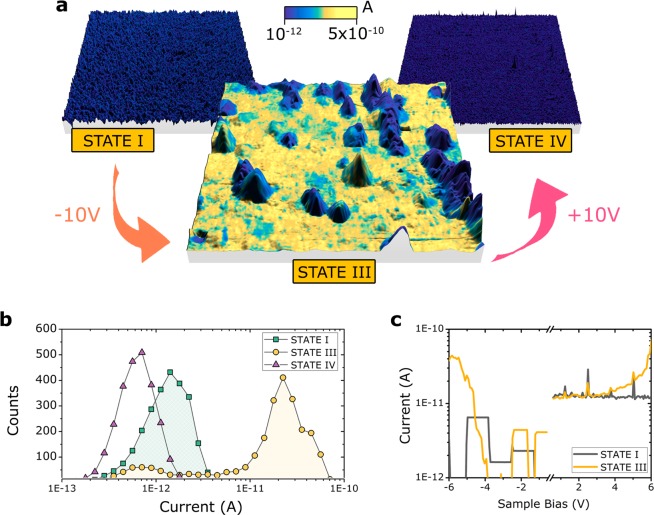
(**a**) 3D representation of the data collected from Conductive Atomic Force Microscopy (C-AFM). The colors represent the recorded current values and the roughness shows the sample topography. (**b**) Pixel histograms from the current data collected in panel (a). (**c**) I-V spectroscopy curves for states **I** and **III** showing the current measured as a function of applied voltage.

If the primary mechanism underlying the magnetic changes observed here are from oxygen migration, then we would also expect to see changes in the electric properties. To probe this, we investigated the electronic properties using Current Atomic Force Microscopy (CAFM) and the results are shown in Fig. 10. We performed a 3D representation of the obtained data, in which the colors represent the current value recorded while the roughness corresponds to the real topography of the sample. From the initial sample, we were not able to record any current flowing through the system, see “State **I**” graph. However, after being treated at −10 V for 120 min, see “State **III**”, we were able to record a current distribution map. We see that the bottom part of the topography is responsible for the change in the current level recorded, while the top topography features remains unaltered. This increase in current flow is consistent with oxygen ion migration causing oxygen redistribution. With a more heterogenous distribution, there should be some areas that are oxygen deficient (*i.e*., have many oxygen vacancies) and are therefore better able to conduct the electrons. Next, the sample was treated at +10 V for 120 min, see “State **IV**”, which returned the current level very close to the initial state-the minimum that our equipment can resolve. This is again consistent with the magneto-ionic mechanism since the positive voltage is expected to return the sample to a more homogeneous oxygen distribution similar to the initial state. Topographically, we do not observe appreciable changes. State I is very flat while state III and IV have some peaks. We believe that there is a bit additional roughness induced by the oxygen migration process which causes these local peaks. Purely topographical images can be found in the Supplementary Information, Fig. S1. From the current data, we performed pixel histograms for each of the three samples, see Fig. 10b. We found that the current level for the −10 V treated sample is almost two orders of magnitude higher than the initial state and +10 V cases which corresponds to a decrease in electronic resistivity. Examples of I-V spectroscopy curves of the initial and −10 V treated sample, as a comparison, in random spots of the surface are shown in Fig. 10c. The reader may note spikes occurring at the exact same position for both states (I and III). These spikes result from an electronic artifact inherent to our measuring conditions. Such spikes should not be interpreted with a physical meaning underneath, instead they should be attributed to changes into the gain value of our variable dynamic range of the transimpedance amplifier (TIA) used.

## Conclusions

Here we have shown that the magnetic properties of cobalt ferrite thin films can be significantly tuned by the application of voltage in an electrochemical cell. The films that were made mesoporous by block copolymer templating showed much larger changes in *M*_*S*_ (with a maximum change of 15% for the porous sample and a maximum change of only 2% for the non-templated sample). We find that by changing the magnitude of the applied field in the range of −1 V to −10 V determines the maximum attainable change in *M*_*S*_. This can be useful for applications such as neural-network inspired computing. Importantly, the change in *M*_*S*_ was semi-permanent and when the negative voltage (−10 V) was removed, the sample magnetization remained 12% higher than initially. A decrease of *H*_*C*_, by a factor ranging from 5% to 28% for applied voltages of −10 V and −50 V, respectively, is also observed. Furthermore, the changes are completely reversible with the application of a positive voltage, which is important for applications such as memory devices. We identified four mechanisms that could potentially explain our results: charge accumulation at the surface (electric double layer), ion intercalation, surface oxidation/reduction reactions and the magneto-ionic effect (O^2−^ ion migration within the films). We then looked critically at the kinetics of the magnetization changes and characterized the change in oxidation state by XPS and the change in lattice spacing by GIXRD. The magneto-ionic effect is the only mechanism that is consistent with all of the data (*i.e*., the only mechanism that explains the lattice spacing changes observed by GIXRD). From this, we conclude that in our studies, the magneto-ionic effect was likely the primary mechanism at work. However, past literature has shown that the magnetic properties of cobalt ferrite can also be affected by ion intercalation, so we suspect that some ion intercalation is also occurring here. Finally, the charge accumulation and oxidation/reduction mechanisms do not explain our results very well but cannot be completely ruled out and may play a minor role here. The large changes in sample magnetization, reversibility of the process and the observed cyclability of the films make this system particularly interesting for a wide range of applications.

## Supplementary information

Supplementary Information
