# Supplementary material for: Reversible, Electric-Field Induced Magneto-Ionic Control of Magnetism in Mesoporous Cobalt Ferrite Thin Films
Source: Sci Rep. 2019 Jul 25;9:10804. doi: 10.1038/s41598-019-46618-6 (PMC6658663; doi:10.1038/s41598-019-46618-6)
Supplement: Supplementary file 1 — Supplementary Information [file 41598_2019_46618_MOESM1_ESM.docx]

**Supporting Information**

**Reversible, Electric-Field Induced Magneto-Ionic Control of Magnetism in Mesoporous Cobalt Ferrite Thin Films**

Shauna Robbennolt,^1^ Enric Menéndez,^1^ Alberto Quintana,^1^ Andrés Gómez,^2^ Stéphane Auffret,^3^ Vincent Baltz,^3^ Eva Pellicer,^1^ Jordi Sort^1,4^

^1^ Departament de Física, Universitat Autònoma de Barcelona, E-08193 Cerdanyola del Vallès, Spain

^2^ Institut de Ciència de Materials de Barcelona (ICMAB-CSIC), Campus UAB, E-08193 Cerdanyola del Vallès, Barcelona, Spain

^3^ SPINTEC, Univ. Grenoble Alpes/CNRS/INAC-CEA, F-38000 Grenoble, France

^4^ Institució Catalana de Recerca i Estudis Avançats (ICREA), Pg. Lluís Companys 23, E-08010 Barcelona, Spain


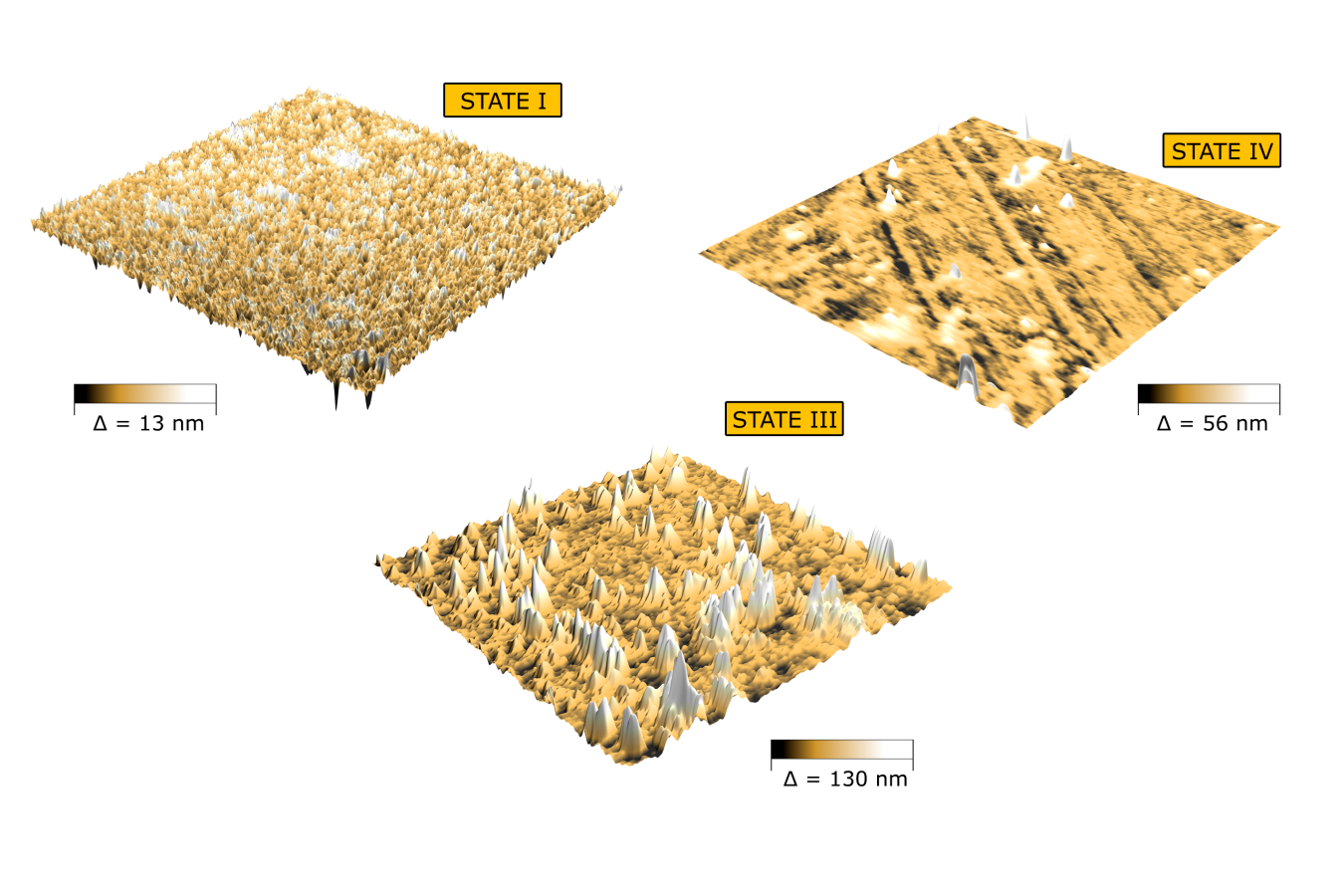


**Figure S1**. Topography images in 3D representation for each of the different State I, II and IV samples.
